# Positive-Pressure Ventilation-induced Pneumothorax After Intubation: A Pandora's Box of Early Diagnostic Pitfalls and Ultrasound-First Management

**DOI:** 10.7759/cureus.91943

**Published:** 2025-09-09

**Authors:** Anindya Dasgupta, Abhradip Das, Swarup Paul, Prasun Banerjee, Bodhisatwa Choudhuri, Siddharth Basu

**Affiliations:** 1 Emergency Medicine, Narayana Multispeciality Hospital, Barasat, IND; 2 Pulmonology, Narayana Multispeciality Hospital, Barasat, IND; 3 Critical Care Medicine, Narayana Multispeciality Hospital, Barasat, IND; 4 Critical Care and Rheumatology, Parkview Super Specialty Hospital, Kolkata, IND

**Keywords:** acute respiratory distress syndrome, artificial respiration, barotrauma, intensive care units, pneumonia, pneumothorax, point-of-care systems, positive-pressure respiration, respirator-induced lung injury, ultrasonography

## Abstract

Pneumothorax under positive-pressure ventilation can present within hours of intubation, particularly when a small, non-recruitable “baby lung” bears most of the mechanical load. We report a 67-year-old man with hypertrophic cardiomyopathy, hypertension, diabetes, and hypothyroidism who arrived obtunded (Glasgow Coma Scale 6) with severe hypoxaemia. He was intubated and initially ventilated in volume control; because saturations remained low with high airway pressures, he was switched to pressure control with higher positive end-expiratory pressure (PEEP). After a brief improvement, he acutely deteriorated with desaturation, hypotension, tachycardia, reduced minute ventilation, and rising airway pressures. Bedside lung ultrasound showed absent sliding with a barcode/stratosphere pattern and a lung point on the right; high-resolution computed tomography (HRCT) confirmed a large right pneumothorax with near-complete right-lung collapse and extensive ipsilateral consolidation. A right intercostal drain produced rapid physiological improvement. Initial studies showed neutrophilic leucocytosis, mild acute kidney injury, a cholestatic-predominant liver profile, markedly elevated NT-proBNP with normal high-sensitivity troponin, and near-normal coagulation; cultures remained negative, and bronchoalveolar lavage GeneXpert and cytology were negative. Endotracheal bleeding with anaemia and thrombocytopenia prompted bronchoscopy, which removed a lower-lobe endobronchial clot. Despite stabilisation, he sustained two intensive care unit (ICU) cardiac arrests with the return of spontaneous circulation; echocardiography demonstrated a dilated left atrium, asymmetric septal hypertrophy with paradoxical septal motion, grade-I diastolic dysfunction, and pulmonary hypertension. Weaning to pressure support occurred on days 4 and 5; he was extubated on day 6, stepped down from ICU on day 7, the chest drain was removed on day 10, and he was discharged home on day 12 on oral antibiotics. At two-week follow-up, he remained stable with no recurrent pneumothorax. This case emphasises three practical points: pneumothorax may occur immediately post-intubation in severely consolidated, low-compliance lungs; ultrasound outperforms supine radiography for rapid bedside diagnosis and should guide timely decompression when physiology is unstable; and power-aware ventilation - limiting driving pressure and avoiding injudicious PEEP escalation in non-recruitable lungs - helps prevent a transient oxygenation “win” from tipping into structural failure.

## Introduction

Pneumothorax remains one of the most feared complications of invasive mechanical ventilation. It commonly accompanies difficult-to-ventilate phenotypes and is associated with longer intensive care unit (ICU) stays and higher mortality [1]. Risk is amplified in heterogeneous, low-compliance lungs - classically severe pneumonia or acute respiratory distress syndrome (ARDS) - where delivered energy concentrates in the small fraction of recruitable “*baby lung*” [2-5]. In ARDS, the "baby lung" refers to the relatively small portion of normally aerated lung available for ventilation, making any given tidal volume proportionally larger [2,3]. "Driving pressure" is the difference between plateau pressure and positive end-expiratory pressure (PEEP) and is a bedside surrogate for cyclic lung stress [5]. "Mechanical power" reflects the energy delivered by the ventilator per unit time (a function of tidal volume, driving pressure, inspiratory flow, and respiratory rate) and relates to ventilator-induced lung injury risk [4,6]. Contemporary physiology reframes “barotrauma” as the downstream result of driving pressure and the mechanical power delivered to the injured lung: tidal volume, PEEP, respiratory rate, flow, and cycling collectively determine stress/strain and thus injury risk [3-5]. Experimental human-relevant work suggests that mechanical power ratio thresholds (e.g., >4.5) and greater exposure time at higher power identify conditions under which ventilator-induced injury - and air leak syndromes - are more likely to emerge [6].

Diagnosis at the bedside is frequently non-trivial. Bedside lung ultrasonography (USG) - also termed point-of-care ultrasound (POCUS) - is now widely recommended in acute respiratory failure to expedite diagnosis and reduce procedure-related complications [7]. Portable supine chest radiographs (CXRs) can miss pneumothoraces, particularly early ones or when air is anterior/apical; by contrast, lung ultrasound provides rapid, radiation-free confirmation through absent pleural sliding, the stratosphere/barcode pattern on M-mode, and the highly specific lung point [7,8]. Computed tomography (CT) remains the anatomic reference, but in unstable patients on positive pressure ventilation, current practice emphasises not delaying decompression when tension physiology is likely [9-11]. Finally, updated guidance on protective ventilation in ARDS underscores careful PEEP/pressure titration in heterogeneous lungs to mitigate the very conditions that precipitate air leaks [2].

Against this backdrop, we report an early post-intubation, positive-pressure-associated large right pneumothorax in a man with multilobar pneumonia and hypertrophic cardiomyopathy, diagnosed ultrasound-first and confirmed on high-resolution computed tomography (HRCT), in whom timely tube thoracostomy and a conservative ventilatory strategy enabled recovery. The objectives of this case presentation are: to demonstrate that (i) higher PEEP can transiently improve oxygenation yet increase mechanical power and precipitate decompensation; (ii) bedside lung ultrasound (POCUS) outperforms supine CXR for rapid diagnosis when a ventilated patient suddenly deteriorates; and (iii) early pleural drainage combined with power-aware ventilation accelerates stabilisation [1-4,6-12].

## Case presentation

A 67-year-old man with hypertrophic cardiomyopathy (HCM), hypertension, type 2 diabetes mellitus, and hypothyroidism was brought to the emergency department (ED) unresponsive. On arrival, he was obtunded (Glasgow Coma Scale (GCS) 6; E1V2M3) with shallow respirations (~10/min) and oxygen saturation by pulse oximetry (SpO₂) 74% on room air. Chest expansion was poor bilaterally with diffuse crepitations and rhonchi; percussion was resonant. Blood pressure measured 140/80 mmHg and peripheral pulses were palpable. There was no preceding fever or cough. Ten days earlier, he had undergone coronary angiography that revealed no obstructive coronary disease.

**Figure 1:**
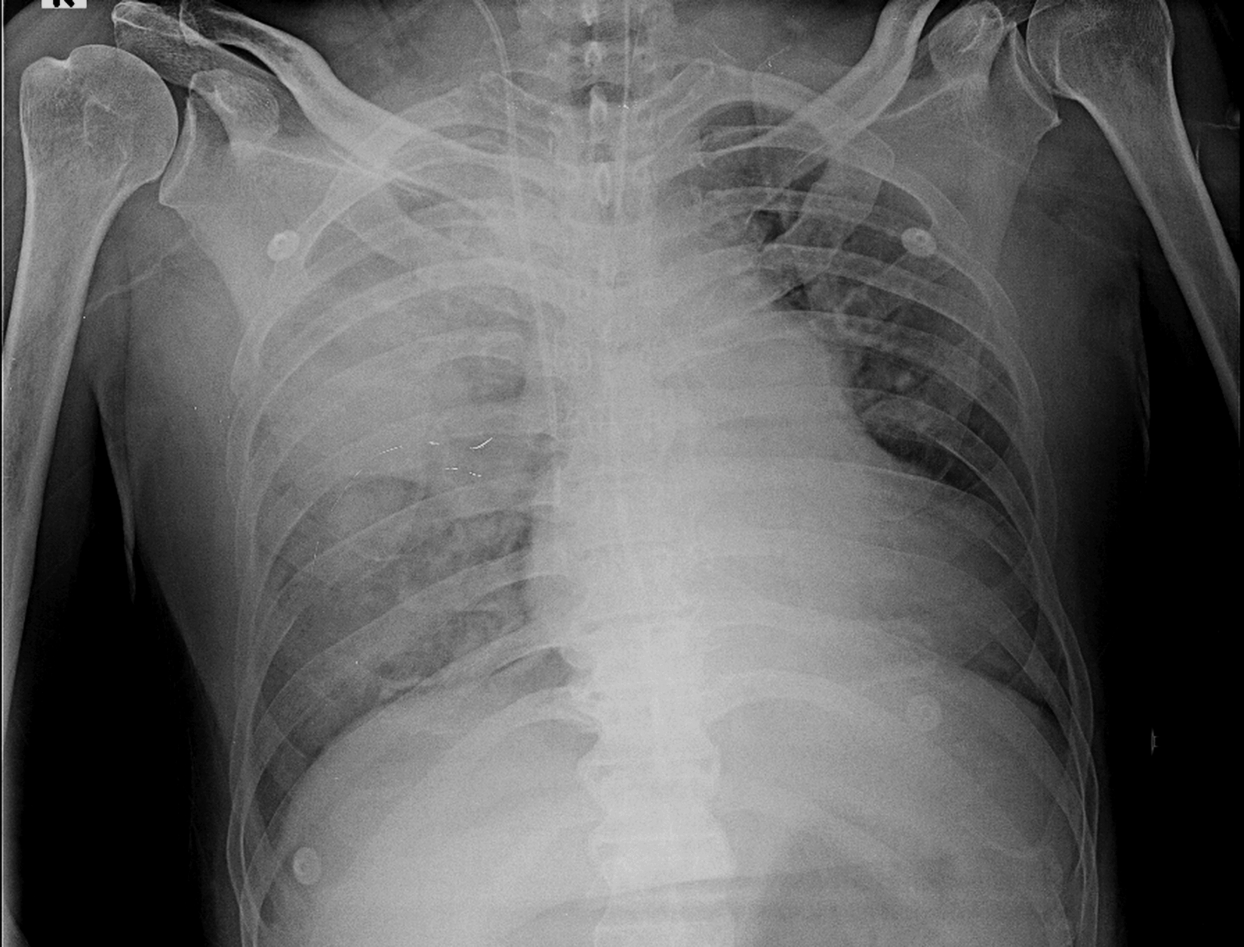
Portable anteroposterior chest radiograph at the emergency department presentation A portable anteroposterior (AP) chest radiograph shows extensive right-sided air-space consolidation. No definite visceral pleural line is identified and there is no radiographic evidence of pneumothorax on this film (noting the reduced sensitivity of portable AP projection). An endotracheal tube is in situ; a central venous catheter is present with the tip projecting over the lower superior vena cava/cavo-atrial junction. External monitoring electrodes are visible.

Because of impending respiratory arrest, the airway was secured with endotracheal intubation and volume-controlled ventilation (VCV) was initiated using lung-protective settings (tidal volume ~6 mL/kg predicted body weight, respiratory rate 18/min). Immediately after endotracheal intubation, a right internal jugular central venous catheter (CVC) was inserted in the ED under real-time ultrasound guidance. A portable anteroposterior (AP) chest radiograph obtained shortly after intubation (Figure 1) confirmed correct endotracheal tube (ETT) and CVC positions, showing extensive right-sided air-space consolidation; no definite pleural line or deep sulcus sign was visible on the supine film. The patient remained haemodynamically stable without respiratory decompensation at this time.

Despite VCV, oxygenation remained marginal and airway pressures were high in a poorly compliant lung. The team therefore switched to pressure-controlled ventilation (PCV) while continuing a lung-protective approach. Immediately after the switch, settings and measured values were: mode PCV; fraction of inspired oxygen (FiO₂), 0.80; PEEP, 10 cmH₂O; inspiratory pressure above PEEP, 12 cmH₂O; tidal volume (Vt), ~390 mL (~6.0 mL/kg predicted body weight (PBW)); respiratory rate (RR), 22 breaths/min; inspiratory-to-expiratory ratio (I:E), 1:2; plateau pressure, 22 cmH₂O; driving pressure, 12 cmH₂O; and static compliance (Cstat), ~30 mL/cmH₂O. Oxygenation improved transiently at these settings (SpO₂ ~94% and arterial oxygen tension (PaO₂) ~78 mmHg on FiO₂ 0.80).

For refractory hypoxaemia, PEEP was subsequently increased. Immediately prior to the event, parameters were: FiO₂, 1.0; PEEP, 14 cmH₂O; inspiratory pressure above PEEP, 16 cmH₂O (Vt ~410 mL, ~6.3 mL/kg PBW); RR, 24 breaths/min; I:E, 1:1.5; plateau pressure, 34 cmH₂O; driving pressure, 18 cmH₂O; and Cstat, ~21 mL/cmH₂O. 

The patient then abruptly deteriorated with acute desaturation and hypotension. SpO₂ fell to 72%-76% on FiO₂ 1.0; heart rate was 136 beats/min; non-invasive blood pressure was 78/46 mmHg (mean arterial pressure (MAP) ~56 mmHg) and end-tidal carbon dioxide (ETCO₂) decreased from ~32 mmHg to ~18 mmHg. The ventilator displayed ‘low exhaled tidal volume’ and ‘low minute ventilation’ alarms. At unchanged pressure-controlled settings, measured exhaled tidal volume fell abruptly from ~400 mL (~6.3 mL/kg predicted body weight) to ~130 mL (~2.0 mL/kg predicted body weight), with minute ventilation decreasing from ~9.1 L/min to ~2.9 L/min. The calculated dynamic compliance dropped to ~8-10 mL/cmH₂O. An end-inspiratory hold was not attempted during the instability; plateau pressure was therefore not measured. 

Bedside lung ultrasound was performed immediately. B-mode imaging showed absent lung sliding over the right anterior chest; M-mode demonstrated a barcode/stratosphere sign, and a lung point was identified laterally - findings strongly supportive of a right pneumothorax. In the ED, given the time-critical deterioration, cine-loop and M-mode clips were not recorded; findings were documented in real time.

**Figure 2:**
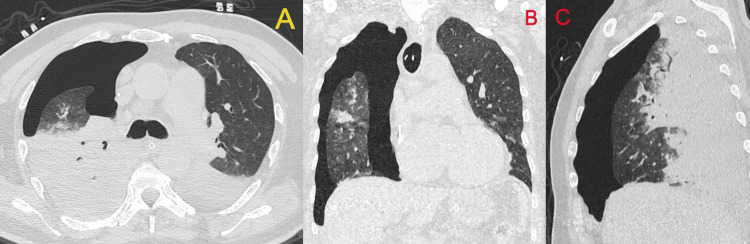
High-resolution computed tomography (HRCT) of the thorax (lung window): large right pneumothorax with compressive atelectasis A (Axial): A large right pleural air collection occupies the anterolateral hemithorax with marked passive collapse of the right lung and dependent basal consolidation with air bronchograms. B (Coronal): The pneumothorax extends from apex to base with a thin right visceral pleural line; the compressed right lung shows multifocal consolidation/ground-glass opacities, while the left lung is relatively spared. C (Sagittal): Sagittal reformat demonstrates the craniocaudal extent of the right-sided pneumothorax and posterior/basal dependent consolidation.

After stabilisation on 100% oxygen and manual ventilation, he was transported for HRCT of the chest immediately. HRCT (Figure 2) confirmed a large right-sided pneumothorax with near-complete collapse of the right lung and confluent consolidation involving the right upper and middle lobes and most of the right lower lobe; lesser inflammatory change was present in the left lower lobe.

**Figure 3:**
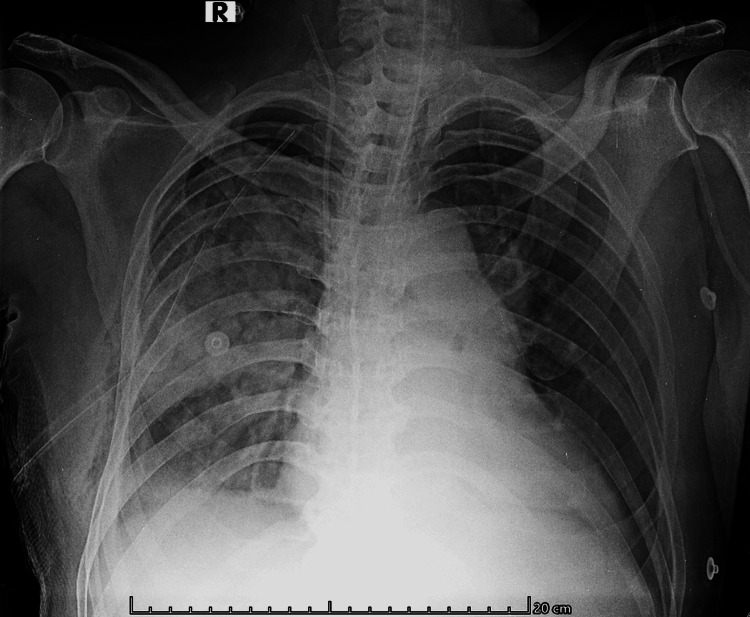
Post–intercostal chest drain portable chest radiograph (anteroposterior view) Portable anteroposterior (AP) chest radiograph obtained after decompression shows a right intercostal chest drain (ICD) coursing cranially along the lateral right hemithorax with the tip projected over the mid-to-upper right chest. An endotracheal tube (ETT) is in situ with the tip above the carina, and a central venous catheter (CVC) is visible. Persistent right-sided air-space consolidation is present with basal volume loss and blunting of the right costophrenic angle, compatible with a small residual effusion. No definite pleural line or deep sulcus sign is seen, indicating no radiographic evidence of a large residual pneumothorax.

A right intercostal drain (ICD) was inserted under ultrasound guidance in the ED, resulting in immediate improvements in ventilator mechanics and gas exchange. SpO₂ rose to 94%-96% on FiO₂ 1.0; blood pressure improved to 104/62 mmHg (MAP ~76 mmHg) with low-dose vasopressor support; ETCO₂ increased to ~30 mmHg; exhaled tidal volume increased to ~345 mL (~5.3 mL/kg predicted body weight) at the same pressure settings; minute ventilation increased to ~7.7 L/min; and dynamic compliance improved to ~17-18 mL/cmH₂O. A post-procedure portable radiograph (Figure 3) confirmed drain position with persistent right-sided consolidation and a hazy right costophrenic angle.

Empiric broad-spectrum antimicrobials were commenced with injection Meropenem 1 g intravenously (IV) every eight hours (adjusted to renal function as needed), injection Teicoplanin with a loading dose of 800 mg IV every 12 hours × 3 doses, followed by 400 mg IV once daily and injection Doxycycline 200 mg IV loading, and then 100 mg IV every 12 hours.

Initial laboratory testing showed neutrophilic leucocytosis, mild acute kidney injury (creatinine 1.49 mg/dL, estimated glomerular filtration rate (eGFR) 47 mL/min/1.73 m²), mild hyponatraemia, and a cholestatic-predominant liver enzyme pattern with hyperbilirubinemia. High-sensitivity troponin I was within the laboratory reference range (2.5 ng/L) while NT-proBNP was markedly elevated (20,700 pg/mL). Coagulation parameters were near-normal. Serial investigations are summarised in Table 1. 

**Table 1:** Serial Laboratory Parameters from Emergency Department Presentation to Pre-discharge (Day 0, Day 5, Day 11–12) Values are shown with institutional reference ranges in the rightmost column. Day 0 indicates Emergency Department (ED) presentation; Day 11–12 indicates the pre-discharge panel. A dash (—) denotes “not assessed.” Abbreviations: ED, Emergency Department; eGFR, estimated glomerular filtration rate; CRP, C-reactive protein; ALP, alkaline phosphatase; AST, aspartate aminotransferase; ALT, alanine aminotransferase; GGT, gamma-glutamyl transferase; A/G ratio, albumin-to-globulin ratio; hs-Troponin I, high-sensitivity troponin I; NT-proBNP, N-terminal pro–B-type natriuretic peptide; FEU, fibrinogen equivalent units; PT, prothrombin time; INR, international normalized ratio; aPTT, activated partial thromboplastin time.

Laboratory parameters	Day 0 (ED presentation)	Day 5	Day 11–12 (pre-discharge)	Normal values
Haemoglobin (g/dL)	9.4	8.3	9.8	14.0–18.0
Total leukocyte count (×10⁹/L)	16.8	7.2	6.8	4.0–10.0
Neutrophils (%)	91.9	78	65	40–80
Lymphocytes (%)	5	15	28	20-45
Platelets (×10³/μL)	145	85	160	150–410
Serum Urea (mg/dL)	58	40	28	17-43
Serum creatinine (mg/dL)	1.49	1.1	0.95	0.66–1.25
eGFR (mL/min/1.73 m²)	47.1	70	85	≥90
Sodium (mmol/L)	133	136	138	137–145
Potassium (mmol/L)	4.9	4.1	3.9	3.5–5.1
CRP (mg/L)	210	80	18	<5
Total bilirubin (mg/dL)	3.3	2	1.2	0.2–1.3
Direct (conjugated) bilirubin (mg/dL)	1.3	0.8	0.4	0.0–0.3
ALP (U/L)	213	180	150	38–126
AST (U/L)	82	45	35	17–59
ALT (U/L)	59	40	32	<50
GGT (U/L)	276	220	180	15–73
Albumin (g/dL)	4.2	3.6	3.8	3.5–5.0
A/G ratio	1.1	1	-	1.0–2.1
Procalcitonin (ng/mL)	3.5	-	0.8	<0.5
hs-Troponin I (ng/L)	2.5	-	-	<10
NT-proBNP (pg/mL)	20,700	8,000	2,500	<125
D-dimer (ng/mL FEU)	1800	920	400	<500
PT (sec) / INR	14.7 / 1.08	-	-	12–16 / 0.8–1.2
aPTT (sec)	34.4	-	-	27–35
Serum lactate (mmol/L)	3	1.6	1.4	0.5–2.0

During the first 24-48 hours, the course was complicated by endotracheal bleeding with a falling haemoglobin and platelet count. Flexible bronchoscopy in the ICU revealed a lower-lobe endobronchial clot, which was irrigated and aspirated with improvement in airway blood burden. He received packed red blood cells and platelet transfusions. Microbiologic studies remained negative: endotracheal suction culture showed no growth and urine culture also showed no growth. Viral and serologic testing for human immunodeficiency virus (HIV), hepatitis B and C, dengue immunoglobulin M (IgM), and leptospira IgM were non-reactive. Bronchoalveolar lavage (BAL) GeneXpert for *Mycobacterium tuberculosis* was negative; BAL cytology showed inflammatory cells with no malignant cells. The illness was therefore classified as culture-negative severe pneumonia/ARDS.

Despite initial stabilisation, his condition worsened in the intensive care unit (ICU) and he suffered two cardiac arrests on separate occasions. Advanced cardiac life support was provided and return of spontaneous circulation (ROSC) was achieved both times. Subsequent two-dimensional echocardiography demonstrated a dilated left atrium, asymmetrical septal hypertrophy of the left ventricle with paradoxical interventricular septal motion, grade I diastolic dysfunction, and pulmonary hypertension with an estimated pulmonary artery systolic pressure (PASP) of 53 mmHg, consistent with the background of HCM and the acute cardiopulmonary stress.

With continued drainage, lung-protective ventilation and supportive care, gas exchange and mechanics improved. VCV/PCV was maintained initially, after which weaning to pressure-support ventilation (PSV) was initiated on days 4 and 5. The patient passed a spontaneous breathing trial and was extubated on day 6 and transferred out of the ICU on day 7. The ICD was removed on day 10 after sustained re-expansion without air leak. On day 12, he was discharged home with a prescription for oral cefuroxime 500 mg twice daily and doxycycline 100 mg twice daily, along with instructions for respiratory physiotherapy. At the two-week outpatient review, he remained well with no recurrent pneumothorax on chest imaging.

## Discussion

Ventilator-associated pneumothorax remains a clinically important complication because it clusters with difficult-to-ventilate phenotypes and tracks with worse outcomes, even in the contemporary ICU era [1,2,13]. What distinguishes this case is the timing: the pneumothorax declared itself within hours of intubation - well before the more typical several-day window reported as disease severity and ventilator exposure accumulate [1,13]. That chronology, set against a background of extensive unilateral consolidation and poor compliance, points less to a cumulative “dose” of ventilation and more to anatomical vulnerability in which a small fraction of aeratable lung - the so-called “baby lung” - absorbs a disproportionate share of the applied forces [3-5]. Contemporary synthesis supports this reframing: recent meta-analytic work estimates pneumothorax around 6%-7% of mechanically ventilated patients while challenging simplistic “high pressure equals barotrauma” narratives and emphasising the integrated concept of mechanical power (the energy delivered to the lung per minute) and its distribution within heterogeneous lungs [2,12].

Physiologically, the patient’s transient improvement in oxygenation after raising PEEP and switching to pressure-controlled ventilation did not alter the underlying geometry of a largely non-recruitable right lung. Injury risk in such settings is better captured by driving pressure and mechanical power than by peak pressure alone; the relevant determinants - tidal volume, PEEP, respiratory rate, flow/cycling - govern the energy per minute delivered to the remaining ventilated units [3-5]. Experimental and clinical data suggest that exceeding proposed mechanical-power thresholds (e.g., a mechanical power ratio >4.5) and time-at-risk above higher power are associated with ventilator-induced lung injury and air-leak syndromes [6,14], a view reinforced by a recent critical review of power-based approaches to ventilator-induced lung injury (VILI) [15]. In our patient, these dynamics plausibly explain the abrupt haemodynamic and ventilatory collapse after PEEP escalation despite the short overall duration of mechanical ventilation, and they are consistent with the rapid physiological recovery after tube thoracostomy.

The temporal sequence and objective data favour ventilator-induced barotrauma over a procedure-related puncture. The right internal jugular CVC was placed immediately after intubation in the ED under real-time ultrasound guidance, and the immediate post-procedure portable chest radiograph (Figure 1) showed no pneumothorax. There was no instability at that time and no interim pleural procedures. The later crash followed escalation of PEEP and inspiratory pressure in PCV, with a concurrent rise in driving pressure and fall in static compliance - an injurious mechanical milieu - collectively supporting a ventilator-induced mechanism [6,12-15].

The diagnostic sequence in this case speaks to practice at the bedside. Supine portable chest radiography is insensitive to anterior or apical pneumothoraces, particularly early and under positive pressure, whereas lung ultrasound can resolve the question in minutes. The combination of absent pleural sliding, a stratosphere/barcode pattern on M-mode, and a lung point carries high specificity for pneumothorax and, when coupled with clinical deterioration, should trigger immediate intervention [7,8]. Ultrasound then remains useful beyond diagnosis; contemporary work supports its use for short-interval monitoring of pneumothorax evolution after an intervention - an approach that limits radiation and shortens decision cycles [16]. In unstable ventilated patients with suspected tension physiology, modern guidance is clear: decompress first and obtain confirmatory imaging later if needed [9-11]. Our patient’s prompt improvement after drainage underscores the point.

Who is at risk deserves emphasis. Severe pneumonia, ARDS and chronic obstructive pulmonary disease (COPD) are repeatedly over-represented among patients who suffer air leaks because low compliance and regional heterogeneity amplify local stress/strain even when global settings appear “protective” [1,5,12,13]. The COVID-19 experience, while pathobiologically distinct, reinforced that lesson at scale: across invasive-ventilation cohorts, barotrauma remained common and signalled higher mortality, despite modern ventilation targets [17]. In addition, large pneumothoraces have been described in ventilated patients with chronic respiratory disease, again highlighting the role of baseline lung vulnerability as the substrate upon which positive pressure precipitates failure [18]. These observations argue for vigilance from the first hours of ventilation rather than only after days of exposure, particularly when raising PEEP to salvage oxygenation in lungs that recruit poorly.

Management choices in our case align with contemporary guidance. The British Thoracic Society (BTS) and the joint European Respiratory Society/European Association for Cardio-Thoracic Surgery/European Society of Thoracic Surgeons (ERS/EACTS/ESTS) recommendations endorse prompt tube thoracostomy in ventilated patients with clinically significant pneumothorax and standardise elements of aftercare; our day-10 removal after sustained re-expansion fits within this framework [9,10]. A 2025 surgical review updates pragmatic details - tube calibre selection, early transition from suction to water seal, and criteria for safe removal - that can streamline recovery and discharge planning [19]. At a policy level, European guidance has evolved toward more conservative pathways for selected spontaneous pneumothoraces, prompting editorials that dub the shift a “Copernican revolution”; while our patient required immediate drainage by virtue of instability and positive pressure, the direction of travel in guideline thinking is still worth noting when framing follow-up and recurrence prevention [10,20]. On the ventilator side, the 2024 American Thoracic Society (ATS) ARDS update situates this case’s trajectory in a broader protective-ventilation strategy: minimise driving pressure, avoid unnecessary PEEP escalation in poorly recruitable lungs, and consider the mechanical-power footprint when balancing oxygenation against injury risk [12].

Taken together, the case illustrates three practical messages that extend the literature. First, timing matters: pneumothorax can appear immediately after intubation in severely consolidated, low-compliance lungs; clinicians should not be reassured by the absence of the “classic” several-day interval. Second, ultrasound outperforms supine radiography when a ventilated patient acutely deteriorates and can guide both immediate decompression and short-interval monitoring. Third, power-aware ventilation - thinking in terms of driving pressure, mechanical power and exposure time - offers a more faithful map of risk than mode labels or peak pressures alone and may prevent a transient oxygenation “win” from tipping into structural failure.

This report has limitations. This single-patient observation from a mixed medical ICU cannot establish a causal link between any specific ventilator setting or manoeuvre and the pneumothorax. High-fidelity ventilator data (continuous waveforms/loops) were not exported. Several values were abstracted from bedside displays and narrative charting. Formal recruitability testing (recruitment-to-inflation ratio, esophageal manometry, electrical impedance tomography, or computed tomography) was not feasible during instability. So transpulmonary pressure and true driving pressure could not be quantified precisely (plateau pressure during instability was also not obtained). We used pragmatic bedside surrogates - oxygenation response, driving pressure, and static compliance. Transient oxygenation gain, rising driving pressure, and falling compliance after PEEP escalation indicated poor recruitability and potential overdistension, which led us to align our management toward decompression and restraint with ‘power-aware’ ventilation rather than further PEEP increments. POCUS was performed in a time-critical resuscitation context; cine-loop and motion-mode clips were not recorded, which may reduce image interpretability despite classic signs (absent sliding, barcode/stratosphere pattern, and a lateral lung point). Microbiologic studies were negative, precluding pathogen-specific conclusions. These constraints limit generalisability but do not alter the central messages of the case - barotrauma occurring early in a low-compliance lung, ultrasound-first recognition of tension physiology, and rapid reversal with timely decompression and power-aware ventilation.

## Conclusions

This case underscores how quickly a positive-pressure-associated pneumothorax can declare itself in a severely consolidated, low-compliance lung - within hours of intubation, not days. When a ventilated patient acutely deteriorates, lung ultrasound (absent sliding, barcode sign, lung point) outperforms supine radiography for rapid decision-making; in the presence of unstable physiology, decompression should not wait for confirmatory CT. Early tube thoracostomy, followed by power-aware ventilation (attention to driving pressure and mechanical power rather than mode labels alone), enabled steady recovery in this patient.

Clinically, the message is simple: patients with pneumonia/ARDS/COPD are at high risk for air-leak syndromes, and escalation of PEEP in non-recruitable lungs can trade a transient oxygenation gain for structural failure. Vigilance during the first hours of ventilation, ultrasound-first diagnostics, and prompt drainage when tension is suspected are the practical safeguards that reduce harm and hasten recovery.
